# The ongoing dissection of the genetic architecture of autistic spectrum disorder

**DOI:** 10.1186/2040-2392-2-12

**Published:** 2011-07-08

**Authors:** Rob F Gillis, Guy A Rouleau

**Affiliations:** 1 Department of Human Genetics, McGill University, Montreal QC, H3A2T5, Canada; 2 Centre of Excellence in Neuromics of Université de Montréal, Centre Hospitalier de l'Université de Montréal Research Center and Department of Medicine, Université de Montréal, Montréal, QC H2L 2W5, Canada; 3 Department of Medicine, Université de Montréal, Montréal, QC, H2L 2W5, Canada

## Abstract

The development of robust, non-hypothesis based case/control studies has led to a large push forward towards identifying common genetic variants that contribute to complex traits. However, despite many attempts, the search for common disease-predisposing variants in childhood developmental disorders has largely failed. Recently, a role for rare causal variants and *de novo *mutations is emerging in the genetic architecture of some of these disorders, particularly those that incur a large degree of selection against the phenotype. In this paper, we examine these data and use classic genetic epidemiological approaches to gain insights into the genetic architecture of ASD. Future studies using next generation sequencing should elucidate the precise role *de novo *mutations play in disorders traditionally thought to have resulted from polygenic or common disease, common variants inheritance.

## Introduction

Autistic spectrum disorders (ASD) are a group of neurodevelopmental disorders clinically characterized by deficits in three core domains termed the phenotypic triad: impairments in social interaction; impairments in communication; and restricted interests and repetitive behavior. The group consists of Asperger's syndrome (AS), pervasive developmental disorder not otherwise specified (PDD-NOS) and the prototypical autistic disorder (AD). They all share a similar age of onset of approximately 12 to 36 months, which corresponds to the developmental time period when spatial and temporal transcriptional cascades lead to remodeling and elaboration of neuronal circuitry [1,2]. The prevalence of ASD is between 10 in 10,000 and 60 to 70 in 10,000, depending on the precise definition used [3]. Evidence from family studies implies that ASD has a strong genetic basis: the concordance rate in monozygotic (MZ) twins ranges from 70 to 90%, whereas dizygotic (DZ) twin concordance varies from 0 to 10% [4,5]. Familial aggregation studies have shown that the relative risk of developing autism in first-degree relatives of an autistic patient is 3 to 7%, which is ten-fold higher than the prevalence in the general population. Although these lines of evidence suggest that the disorder is primarily of genetic origin, the genetic susceptibility factors responsible for ASD have remained largely elusive, despite several recent advancements in the field.

Despite the seemingly precise definition of ASD above, it must be understood that ASD is not necessarily a simple binary diagnosis. ASD is a grouping of childhood developmental disorders that reflect the difficulties in categorizing psychiatric disorders on the background of a shifting landscape of child development, neuroplasticity and historical nosology, which do not necessarily reflect a homogenous grouping of phenotypes with shared genetic characters on the underlying biological level. ASD has a wide clinical spectrum, with some individuals being high-functioning college students with above-average IQs [6], a fraction being able to perform astonishing memory feats and calculations [7], and others being non-verbal with severe mental retardation, involving self-injury [8]. Comorbidity with epilepsy is estimated at approximately 30% [9,10], while a strict definition of macrocephaly is seen in approximately 20% of children diagnosed with ASD [11].

Although recent studies have highlighted that *de novo *mutations and copy-number variations (CNVs) may be involved in a significant proportion of ASD cases [12-21], identifying whether the remaining cases of idiopathic ASD result from common variants with low effect sizes or rare variants/*de novo *mutations with high penetrance is important to help direct and guide future research and funding efforts aimed at gene identification. Although obtaining precise, multigenerational genome-wide sequence information is not currently plausible, it is still possible to examine the empirical data that are directly influenced by the underlying genetic architecture to deduce the potential contributions that *de novo *mutations may provide to the overall proportion of idiopathic ASD cases.

It has been well established that large-scale *de novo *chromosomal anomalies frequently occur in patients with ASD, explaining approximately 5 to 10% of cases [22,23], including reports showing a strong association with *de novo *CNVs [18]. It is also believed that techniques providing higher resolution of CNVs will probably identify smaller *de novo *CNVs responsible for an even higher overall proportion of ASD cases [24].

To date, the mutations discovered in genes that have been associated with high penetrance and risk for displaying the ASD phenotype are those that have arisen *de novo *with limited transmission, as reviewed previously [25]. In terms of function, 13 of 26 high-risk high-penetrance mutations have been discovered in genes expressed at the synapse [25]. Others have also been described in transcription factors expressed in the fetal brain (in the case of *ARX *[26]), genes involved in translational repression by RNA binding (in the case of *FMR1*, the gene responsible for fragile X syndrome[27-29]), and in tumor suppressor genes (for example, *TSC1 *and *TSC2*) [28,30,31]. Although there a higher proportion of mutations have been found in genes expressed at the synapse, the results do not indicate a convergence on any particular pathway or molecular machinery that will homogenously distinguish patients currently labeled on the phenotypic level with ASD. Therefore, ASD represents monogenic dysfunction in a vast array of molecular pathways and functional structures, all leading to the same general phenotype, implicating possibly hundreds of potential candidate genes. For example, using conservative numbers, there are approximately 3,000 genes expressed at the synapse that contribute to synaptic function. Countless more are brain-expressed embryonic transcription factors, translational repressors and tumor suppressors. Certainly, mutations in all of these candidate genes and their downstream targets are unlikely to produce the ASD phenotype; however, there is clearly a potential for the same phenotype to arise as a result of monogenic mutations in any one of several hundred genes.

Although not all amino acid substitutions will have a functional effect, a significant fraction will lead to disease. It has been estimated that in every zygote, there are approximately 1 to 3 new deleterious mutations that lead to an altered amino acid per genome, this is on average 1 new mutation per 10,000 genes/zygote [19,32]. Therefore, for a disease such as ASD that may result from dysfunction in any one of hundreds of different genes, new mutations may be responsible for a significant fraction of cases. Examining conditions for which genes have previously been identified, it is evident that new mutations are common. For example, 1 in 6,000 live births harbor a novel mutation causing neurofibromatosis type 1 (NF1) [33,34]. The frequency of new point mutations in Duchenne muscular dystrophy is similar: 1 in 10,500 live births [35]. These are large genes allowing for a high mutation rate; however, their total genomic size is a small fraction compared with the genomic size of the hundreds of potential genes that may all produce the same general ASD phenotype. The estimated mutation rate of a repetitive stretch of genomic DNA is approximately 100,000 times more frequent than common point mutations [36]. These stretches can code for poly-amino acid tracts, which are found in hundreds of genes throughout the genome, or for regulatory elements such as *FMR1*. For example, alanine tract expansions in the *ARX *gene, a transcription factor expressed in the fetal brain, produces a broad spectrum of disorders including epilepsy, mental retardation and the ASD phenotype [26,37]. In the case of *FMR1*, an expansion of the repeat within the upstream regulatory region leads to hypermethylation and silenced gene expression [38]. Given the ubiquitous presence of repetitive stretches of DNA within coding regions or regulatory genetic elements, *de novo *expansions or contractions of these repetitive elements could account for a fraction of ASD cases as well.

Taken together, these above examples show that *de novo *mutations could potentially occur with a sufficiently high frequency to explain the relatively high incidence of ASD, and act dominantly to do so. Mutations in known genes for ASD currently explain only a small fraction of cases. Under a model dominated by *de novo *mutations, over time more monogenic mutations should be discovered in different genes, each contributing a small portion of the overall disease incidence. The question is whether a model such as this can be reconciled with all of the data gleaned from genetic epidemiologists to account for the genetic architecture of ASD.

One of the predictions of an architecture dominated by *de novo *mutations implies that individuals diagnosed with ASD are at increased risk for a recurring phenotype in their children, owing to the transmission of the recently arisen dominant mutation. Although such a study is difficult to undertake, given the low reproductive rate of individuals with ASD, a study investigated this prediction by examining multiplex families with idiopathic forms of ASD from the Autism Genetic Resource Exchange (AGRE) database [12]. They found that families with unidentified mutations can be grouped into two types: a small minority for whom the risk of autism in male offspring is near 50%, and the vast majority, for whom male offspring have a low risk. They proposed that sporadic autism in the low-risk families is mainly caused by spontaneous *de novo *mutations, with high penetrance in males and relatively poor penetrance in females. They explained that those high-risk families include offspring, most often unaffected females, who carry a new dominant mutation, and in turn transmit the mutation to their offspring. Obviously, by looking at cases in multiplex families, which represent a small fraction of all cases of autism, these conclusions cannot be generalized. A more relevant study, looking at the children of sporadic cases of autism, has not been reported.

### Twin studies and sibling recurrence

As mentioned above, the MZ twin concordance rate for ASD is 70 to 90% and the DZ concordance is similar to the sibling recurrence rate of 0 to 10%, with the extreme values probably being representative of smaller sample sizes [4,5,39]. Given these numbers, there are two possible explanations for the data: a polygenic model involving several genes interacting with environmental factors, or *de novo *mutations with limited transmission. Most *de novo *mutations will occur in affected individuals and will not be transmitted; however, they can be transmitted from unaffected parents to multiple affected siblings as a result of maternal transmission of mutations on the X chromosome to male offspring [40,41] or of gonadal mosaicism [24,42], which together would result in higher sibling recurrence rates. Furthermore, it is also theoretically possible for transmission to occur from asymptomatic parents via autosomal recessive inheritance patterns or through mutations in imprinted genes. Although features of ASD have been identified in other syndromic epigenetic disorders [43], ASD-specific examples of highly penetrant genetic variants whose transmission was enabled by unaffected family members via imprinting have yet to be discovered.

Although perfect concordance between autistic MZ twins is not seen, this does not necessarily imply that environmental factors must play a role in producing or preventing the ASD phenotype. Discordance could occur as a result of variable X-linked inactivation [44], somatic mosaicism for a *de novo *mutation that occurred early in development in only one of the two developing zygotes, or autosomal dominance with variable expressivity, potentially resulting from stochastic events during embryogenesis.

### Association studies/linkage studies

To date, there have been three major genome-wide association studies (GWAS) aimed at identifying common variants that predispose to ASD. The initial study investigated 780 families (3101 subjects) and a second cohort with 1204 affected individuals (all of European ancestry) and identified significant association of six single-nucleotide polymorphisms (SNPs) with ASD to chromosome 5p14, an intergenic region between cadherins 9 and 10. These genes are involved in neuronal cell adhesion, and could be viewed as promising candidates [45]. The second study involved a combined linkage and association study of 1031 families. Although no promising linkage results were discovered, an association to an SNP near the gene *SEMA5A *was shown, and this was combined with expression analysis detailing decreased expression of this gene in the brains of patients with ASD [46]. The third study, comprising 1558 individuals found genome-wide evidence for association at the *MACROD2 *locus, although the authors had difficulty maintaining this signal in a replication cohort [47].

Notably, despite the robust sample sizes of these studies, none of these groups were able to replicate each other's results and each of the studies found modest effect sizes ranging from 0.55 to 1.2. Furthermore, these studies were carried out on modern platforms containing tagged SNPs for common CNVs, therefore it is also unlikely that such CNVs will explain an appreciable fraction of idiopathic ASD [48].

There have also been a countless number of candidate gene association studies performed in the past, which have suffered from the same curse of inconsistent replication. Despite these repeated efforts, there are still no common variants that could be inarguably associated with ASD in the same manner in which common variants have recently been identified for other complex traits (it should be noted that successful association studies have generally studied traits in which little selection occurs against the phenotype). If the genetic architecture of ASD is dominated by *de novo *mutations, followed by strong selection against the phenotype, it is clear that association studies will never identify clear signals, as these causal mutations will never have time to establish themselves in linkage disequilibrium with SNPs on a genotyping platform at a population-wide level. However. it is theoretically possible for an association to develop between a common variant and a region prone to a non-homologous allelic recombination that could lead to the ASD phenotype. Although abstract, this is not an unprecedented example [49-54]. Overall, the lack of replication in these association studies would indicate that if common variants are still responsible for a significant fraction of idiopathic ASD, the number of common variants involved would have to be relatively high and their effect sizes would have to be considerably small, as has been recently shown[55].

There have also been numerous non-parametric linkage studies combining multiple small families, which have produced inconsistent results, with linkage peaks over the entire genome [56,57]. The benefit of non-parametric linkage is that this method should also identify rare variants or transmitted mutations on different haplotypes at a small number of loci. A problem would arise under an architecture dominated by *de novo *mutations on a genome-wide level, as most sample cohorts would be biologically and genetically heterogeneous. Chromosomal regions of haplotype or allele sharing between siblings will be very large, therefore highly penetrant shared haplotypes between siblings could potentially be uniformly distributed throughout the genome in any ASD cohort, resulting in random noise during linkage analysis.

### Consistent global prevalence

Obtaining global disease incidence data for ASD is difficult, as diagnostic disparities coupled with little clinical investigation of ASD in certain regions is a hindrance. However, studies have failed to conclusively reveal an uneven distribution of ASD from any ethnic background [3]. One of the many perplexing aspects of ASD is its relatively high frequency and high heritability, despite strong selection against the phenotype. This might be explained by strong selection for disease-predisposing alleles, as is seen for example in sickle-cell disease. However, it would be expected that such a strong selective pressure would be related to specific environments, which differ in different parts of the world, and so would predict an uneven global distribution, which is not the case for ASD. Under a polygenic model, this could only be reconciled by the existence of many allelic variants with low effect sizes that confer a susceptibility to ASD dating back to a common founder population for all of humanity. These variants would have had to be spread globally through small human populations as they migrated to various locations around the world. Information gleaned from the International Hapmap project has shown that a considerable amount of human genetic variation is common between several populations around the globe, indicating that a substantial proportion of variants date back to a common founder population [58]. Therefore, on the surface, this concept is plausible.

In small isolated founder populations, such as those likely to be found in many of our ancestral migratory movements, the genetic variation is reduced, with a concomitant increase in homozygosity at all loci, as founder effects coupled with genetic drift act to reduce overall genetic variation. This would posit that under a polygenic model, the allelic variants that produce the ASD phenotype must have combined more frequently to produce individuals that would be modernly diagnosed as autistic. In order for these variants to have remained globally at a similar frequency, there could not have been any selection acting against them, or any fixation by genetic drift during small population migrations and settlements, otherwise an uneven disease distribution would be presently observed.

In our modern environment, autistic individuals frequently survive into adulthood, yet strong negative selection still occurs against the phenotype, as evidenced by their low reproductive rates [59]. A gene pool is a reflection of successful reproductions between ancestral genomes that were composed of allelic variants whose collective actions conferred advantages to their historical possessors. The persistence of allelic variants under negative selection throughout evolutionary history in all human populations around the globe disagrees with evolution at the genetic level, and requires an explanation. Considering the low transmission of the ASD-conferring allelic variants, it is thus very unlikely that the remaining cases of idiopathic ASD could explain an even disease distribution around the globe.

Analyzing this observed feature under the genetic architecture of idiopathic ASD dominated by *de novo *mutations, an even disease distribution would be predicted, as mutation rates should not be considerably different between any specific global populations.

### Skewed male:female ASD ratio

It is already known that males are more prone to developing the autistic phenotype than females, by a ratio of approximately 4:1 [60]. It has been suggested that intrinsic differences between male and female brains may be a reason why one sex is more vulnerable to the ASD variants [61]. This explanation may apply equally well to a *de novo *hypothesis as a polygenic model. A previous study highlighted that in high-risk families, males are more likely than their female relatives to inherit the condition, despite their shared genetic background [12]. The selective advantage of diploidy, as a result of its protective mechanism against *de novo *mutations, is intuitive, and it would therefore follow that hemizygous males would be prone to the effect on the X chromosome. There is also evidence that the X chromosome has a higher proportion of genes involved in brain development and cognition than of autosomal genes [62,63]. A recent review highlighted that 6 of 26 genes that show evidence of causal, monogenic dysfunction in non-syndromic ASD are located on the X chromosome [64], which might be considered too low a number to fully explain the skewed sex ratio, given the degree of scrutiny the X-chromosome has received [65]. However, it must be noted that defining the precise number of currently identified ASD alleles can be difficult, given the degree of variable expressivity in neurodevelopmental disorders and the degree of clinical overlap between ASD and other known syndromic conditions. Using another recent review, which developed a list containing every gene identified in ASD and all conditions that currently share a clinical overlap with features of ASD, the proportion of genes on the X chromosome was listed at 45 of 103 [66]. Whether females are resistant to contributing high-penetrance autosomal variants or whether a greater proportion of X-chromosome mutations will be discovered must be left for future research. It should also be noted that *de novo *mutations occurring on the X chromosome in females can be briefly transmitted to produce affected males, unless unfavorable X-chromosome inactivation produces a phenotype in females. However, it would be rare for the transmission to occur through multiple generations, as the penetrant mutation is unlikely to be transmitted by male offspring.

### Presence of autistic features in first-degree relatives

There have been many studies showing that first-degree relatives of individuals diagnosed with ASD can often show some of the broader phenotypic traits of autism [67,68]. These studies were carefully conducted with proper controls, and were able to achieve statistical significance consistently.

A polygenic model would nicely account for this finding, proposing that allelic ASD variants for every autistic trait circulate in the population. The child diagnosed with ASD would be considered unfortunate to have acquired by chance a higher number of these allelic variants, which caused them to surpass the threshold in the phenotypic triad. The existence and transmission of harmful allelic variants through multiple generations has been discussed in a previous section. It has also been suggested that the allelic variants predisposing to the phenotypic triad are inherited separately [69]; however, this poses a perplexing question: how do the allelic variants predisposing to the phenotypic triad tend to co-occur with such high frequency? The existence of numerous tightly linked haplotypes located throughout the genome, containing clusters of ASD-conferring allelic variants that have evaded natural selection, is unlikely. There could be an unknown mechanism that leads to this higher than expected co-segregation, or perhaps even a synergistic relationship between them. However, mutations in several genes have already been discovered whose global brain expression results in global deficits in all three domains [13,40,41,70], which questions the necessity of investigating this avenue.

Under an architecture dominated by *de novo *mutations, the examples of clearly identifiable, carefully controlled observation of milder forms of autistic symptoms in first-degree relatives could result from X-linked inactivation in females, as well as autosomal dominant inheritance with variable expressivity. X-linked inactivation will produce a diverse array of phenotypes in female patients, dependent on the specific location of inactivation of the wild-type chromosome. It is easy to imagine the implications of this if stochasticity resulted in the inactivation of the unaffected chromosome in only a portion of the brain of female carriers. This would conceivably result in various phenotypes, depending on the function of the gene and the proportion and location of the expression of the mutant copy.

Autosomal dominant mutations in genes that are involved in embryonic development can result in an incredibly diverse array of phenotypes. Mutations in the Sonic Hedgehog protein (SHH), for instance, result in holoprosencephaly, a condition whose behavioral and cognitive phenotype can be similar to ASD. Mutations in this gene can result in dramatically different phenotypes, from cyclopia in one family member to slight midline abnormalities in another [71]. If the phenotype of mutant SHH-associated holoprosencephaly were defined on narrowed thresholds, there would undoubtedly be pure cases of holoprosencephaly, with siblings showing the broader spectrum of holoprosencephalic features. The mechanism that leads to a phenotype with variable expressivity at most loci is unknown; it is therefore possible that other genetic variants and environmental factors may influence the ASD phenotype when such a gene is mutated.

## Conclusion

Given the diverse nature of the ASD phenotype, it would be foolhardy to attempt to provide an absolute cause for the disorder, whether from the perspective of neuroscience, psychology or genetics. Therefore, an architecture for ASD implying a causal role for *de novo *mutations is not mutually exclusive to that of a model with common variants. However, as the dissection of this phenotype has progressed, it has become clear that the role of common variants in the phenotype is far lower than initially predicted.

Although ASD represents a continuum on the phenotypic level, this does not necessarily imply the presence of a continuum at the genetic level. It is possible that numerous monogenic, discrete genetic mutations can produce a large spectrum of inextricable phenotypes. This would make it almost impossible to create phenotypic subsets of patients corresponding to the underlying genetic mutation, even with the conceit of hindsight provided after the genetic mutation is identified in a particular patient. Evidence has already shown that *de novo *mutations play a role in the development of ASD; the current remaining question is whether a *de novo *mutation model underlies the majority of the remaining cases of idiopathic ASD.

## Competing interests

The authors declare that they have no competing interests.

## Authors' contributions

RFG wrote and prepared this article. GAR revised, examined and funded the article. Both authors have read and approved this manuscript.
